# Spanish translation of the International Glossary on Infertility and Fertility Care, 2017

**DOI:** 10.5935/1518-0557.20230010

**Published:** 2023

**Authors:** M. Belén Herrero, Clara I. Marín-Briggiler, María Graciela Alaluf, Gustavo Martinez, Gustavo M. Estofan, SAMeR (Argentinian Society of Reproductive Medicine), Buenos Aires, Argentina

**Affiliations:** 1 External reviewer of the Accreditation Program for Medically Assisted Reproduction Institutions, SAMeR - Research Officer, McGill University Health Centre, Montreal, Canada; 2 Research scientist, Instituto de Biología y Medicina Experimental (IBYME-CONICET) - Auditor of Accreditation Program for Medically Assisted Reproduction Institutions, SAMeR, Buenos Aires, Argentina; 3 Assessment coordinator of the Accreditation Program for Medically Assisted Reproduction Institutions, SAMeR - External consultant for health medical institutions, Buenos Aires, Argentina; 4 Director of the Accreditation Committee for Medically Assisted Reproduction Institutions, SAMeR - Vice-president of the Latin American Network of Assisted Reproduction (RedLARA) - Professor, Belgrano University, Buenos Aires, Argentina; 5 President of SAMeR - Member of the Accreditation Committee for Medically Assisted Reproduction Institutions, SAMeR - Regional Director of the Latin American Network of Assisted Reproduction (RedLARA)

**Keywords:** assisted reproductive technology, fertility care, glossary, ICMART, Spanish

## Abstract

In December 2014, the International Committee for Monitoring Assisted Reproductive Technology under the umbrella of the World Health Organization convened an expert meeting to re-examine, update and expand the infertility glossary previously published in 2009. Thus, the International Glossary of Infertility and Fertility Care was developed and published in 2017 simultaneously in Fertility and Sterility and Human Reproduction. In this article, we present the glossary translated into Spanish, obtained after evaluation by Argentinian experts in the field of assisted reproductive technologies, reviewed by Dr. Zegers-Hochschild and approved by the board of the Argentinian Society of Reproductive Medicine (SAMeR). The translation of the glossary to Spanish will facilitate communication between professionals responsible for the practice of ART in Spanish-speaking communities. Moreover, it will lend support to promote better understanding as well as safer and better care for Spanish-speaking minorities and those experiencing cross-border reproductive care.

The International Committee Monitoring Assisted Reproductive Technologies (ICMART) is an independent, international non-profit organization that has taken a leading role in the development, collection and dissemination of worldwide data on assisted reproductive technology (ART). Regional and national registries, such as the European Society of Human Reproduction and Embryology (ESHRE), the Society for Assisted Reproductive Technology (SART) and the Latin American Network of Assisted Reproduction (REDLARA), report their results to ICMART. Thus, in an effort to synchronize the reporting, ICMART in collaboration with the World Health Organization (WHO) compiled a standard set of definitions on ART published in 2006 (Zegers-Hochschild *et al*., 2006). The glossary was then updated in 2009 (Zegers-Hochschild *et al*., 2009) and translated into Spanish (WHO, 2022), Dutch (De Neubourg *et al*., 2012) and Russian (WHO, 2022). Since new techniques become available and corresponding clinical policies are introduced, ICMART together with WHO convened a meeting of experts in 2014, to update and expand the infertility glossary previously published in 2009 (Zegers-Hochschild *et al*., 2009). Between 2014 and 2017, a group of 25 international experts worked on a proposal consisting of 283 terminologies, which was agreed upon by 108 embryologists, fertility experts, public health experts, researchers, and representatives of civil societies and other international organizations.

The International Glossary of Infertility and Fertility Care, 2017 is ultimately the result of a work led by ICMART together with a worldwide consortium of international organizations related to reproductive health: the American Society for Reproductive Medicine (ASRM), the European Society of Human Reproduction and Embryology (ESHRE), the International Federation of Fertility Societies (IFFS), March of Dimes (MOD), the African Fertility Society (AFS), the Groupe Interafricain d’Etude de Recherche et d’Application sur la Fertilité (GIERAF), the Asian Pacific Initiative on Reproduction (ASPIRE), the Middle East Fertility Society (MEFS), the Latin American Network of Assisted Reproduction (REDLARA), the International Federation of Gynecology and Obstetrics (FIGO). It was agreed with the corresponding editors to have the glossary published simultaneously in the journals Fertility and Sterility (Zegers-Hochschild *et al*., 2017a) and Human Reproduction (Zegers-Hochschild *et al*., 2017b) due to the increasing need for an efficient and effective communication among the ART professional community.

The current article contains a translation into Spanish of the 283 terms and definitions described in the ICMART 2017 glossary (Table 1) (Zegers-Hochschild *et al*., 2017a;b). The translated text was performed by specialists in the field of ART and sent for feedback and revision to a working group established by the board of SAMeR. In order to facilitate international communication, the acronyms have remained in its original language. Furthermore, the titles in English have been kept together with the title translated to Spanish, in order to make it easier for professionals to know where each definition comes from in its original form. After approval, permission for publication was requested to the journal Fertility and Sterility.

**Table 1:** ICMART terms translated to Spanish. The acronyms remained in English.

Término en español (Spanish term)	Definición en español (Spanish definition)	Término en inglés (English term)
Aborto espontáneo	La pérdida espontánea de un embarazo intrauterino antes de las 22 semanas completas de edad gestacional.	Spontaneous abortion/miscarriage
Aborto espontáneo recurrente	Pérdida espontánea de dos o más embarazos clínicos antes de las 22 semanas completas de edad gestacional.	Recurrent spontaneous abortion/miscarriage
Aborto espontáneo retenido	Pérdida espontánea de un embarazo clínico antes de las 22 semanas completas de edad gestacional, en la que el/los embrión/embriones o feto/s es/son inviable/s y no es/son espontáneamente absorbido/s o expulsado/s del útero.	Missed spontaneous abortion/missed miscarriage
Aborto inducido	Pérdida intencionada de un embarazo intrauterino, mediante la intervención de medios médicos, quirúrgicos o no especificados (véase reducción embrionaria/fetal inducida).	Induced abortion
Acrosoma	Estructura unida a una membrana que cubre la parte anterior de la cabeza del espermatozoide y que contiene las enzimas necesarias para penetrar en la zona pelúcida del ovocito.	Acrosome
Activación partenogenética	Proceso por el que un ovocito se activa para comenzar el desarrollo en ausencia de fecundación.	Parthenogenetic activation
Adenomiosis	Forma de endometriosis marcada por la presencia de un epitelio similar al endometrio y un estroma fuera del endometrio, en el miometrio.	Adenomyosis
Adherencias	Bandas de tejido cicatricial fibroso que pueden unir los órganos abdominales y pélvicos entre sí, incluidos los intestinos y el peritoneo. Pueden ser densas y gruesas o blandas y finas.	Adhesions
Aglutinación	Agrupación de espermatozoides en el eyaculado.	Agglutination
Aislamiento de espermatozoides	Procedimiento que consiste en la separación de los espermatozoides mediante centrifugación y resuspensión en medios de cultivo. Puede utilizarse para eliminar el plasma seminal y los agentes infecciosos antes de los procedimientos de inseminación intrauterina (IUI) y técnicas de reproducción asistida (ART). Este procedimiento ha demostrado ser eficaz en la eliminación del virus de la inmunodeficiencia humana (HIV). También puede ser eficaz para eliminar otras partículas infecciosas, pero la seguridad y la eficacia clínicas deben establecerse para cada infección particular. Este término se denomina a veces “lavado de espermatozoides”.	Sperm isolation
Análisis del semen	Evaluación del eyaculado para determinar el funcionamiento del aparato reproductor masculino. Los parámetros característicos son el volumen, el potencial de hidrógeno (pH), la concentración, la motilidad, la vitalidad, la morfología de los espermatozoides y la presencia de otras células.	Semen analysis
Andrología	La práctica médica que se ocupa de la salud del sistema reproductor masculino.	Andrology
Aneuploidía	Un número anormal de cromosomas en una célula. La mayoría de los embriones con aneuploidías no son compatibles con la vida.	Aneuploidy
Aneuploidías complejas	Dos o más aneuploidías que afectan a diferentes cromosomas del embrión. Cuando ocurre en los autosomas, esta condición no es compatible con la vida humana.	Complex aneuploidies
Anomalías congénitas	Trastornos estructurales o funcionales que se producen durante la vida intrauterina y que pueden identificarse antes del nacimiento, al nacer o más adelante. Las anomalías congénitas pueden ser causadas por defectos de un solo gen, trastornos cromosómicos, herencia multifactorial, teratógenos ambientales y deficiencia de micronutrientes. Debe informarse el momento de la identificación.	Congenital anomalies
Anomalía congénita mayor	Anomalía congénita que requiere la reparación quirúrgica de un defecto estructural o funcional evidente o que pone en peligro la vida, o causa la muerte.	Major congenital anomaly
Anticuerpos antiespermáticos	Anticuerpos que reconocen y se unen a los antígenos de la superficie del espermatozoide.	Anti-sperm antibodies
Asesoramiento sobre infertilidad	Una intervención profesional con la intención de mitigar las consecuencias físicas, emocionales y psicosociales de la infertilidad.	Infertility counseling
Aspermia	Falta de eyaculación externa.	Aspermia
Aspiración de ovocitos	Aspiración de los folículos ováricos realizada con el objetivo de recuperar ovocitos.	Oocyte aspiration
Aspiración/extracción microquirúrgica de espermatozoides de epidídimo (MESA/MESE)	Procedimiento quirúrgico realizado con la ayuda de un microscopio quirúrgico para recuperar espermatozoides del epidídimo de hombres con azoospermia obstructiva. En ausencia de aumento óptico, cualquier procedimiento quirúrgico para recuperar espermatozoides del epidídimo debe registrarse también como MESE.	Microsurgical epididymal sperm aspiration/extraction (MESA/MESE)
Aspiración/extracción testicular de espermatozoides (TESA/TESE)	Procedimiento quirúrgico que implica una o más biopsias testiculares o aspiraciones con aguja para obtener espermatozoides para su uso en fertilización *in vitro* (IVF) y/o inyección intracitoplasmática de espermatozoides (ICSI).	Testicular sperm aspiration/extraction (TESA/TESE)
Aspiración percutánea de espermatozoides de epidídimo (PESA)	Procedimiento quirúrgico en el que se introduce una aguja por vía percutánea en el epidídimo a fin de obtener espermatozoides.	Percutaneous epididymal sperm aspiration (PESA)
Astenoteratozoospermia	Porcentajes de espermatozoides mótiles y morfológicamente normales en el eyaculado por debajo del límite de referencia. Al informar los resultados, deben especificarse los valores de referencia.	Asthenoteratozoospermia
Astenozoospermia	Porcentaje de espermatozoides mótiles en el eyaculado por debajo del límite de referencia. Al informar los resultados, deben especificarse los valores de referencia.	Asthenozoospermia
Atención reproductiva transfrontera o entre países	La prestación de servicios de salud reproductiva en una jurisdicción diferente o fuera de un país dentro del/de la cual la/s persona/s reside/n legalmente.	Cross-border reproductive care
Ausencia bilateral congénita de los conductos deferentes (CBAVD)	La ausencia, al nacer, de ambos sistemas de conductos (vasos deferentes) que conectan los testículos con la uretra y puede estar asociada a la mutación del gen regulador de la conductancia transmembrana de la fibrosis quística (CTFR). Aunque los testículos suelen desarrollarse y funcionar con normalidad, los hombres presentan azoospermia.	Congenital bilateral absence of the vasa deferentia (CBAVD)
Ausencia de hijos	Condición en la que una persona, voluntaria o involuntariamente, no es padre/madre legal o socialmente reconocido/a, o todos sus hijos han muerto.	Childlessness
Ausencia primaria de hijos	Condición en la que una persona nunca ha dado a luz a un niño vivo, o nunca ha sido padre/madre legal o socialmente reconocido/a de un niño.	Primary childlessness
Ausencia primaria e involuntaria de hijos	Condición de una persona que con deseos de tener hijos, nunca ha dado a luz a un niño vivo, o nunca ha sido el padre/la madre legal o socialmente reconocido/a de un niño. Una de las principales causas de la ausencia primaria e involuntaria de hijos es la infertilidad.	Primary involuntary childlessness
Ausencia secundaria e involuntaria de hijos	Condición de una persona con deseo de tener hijos, que previamente ha dado a luz a un niño vivo, o es/ha sido padre/madre legal o socialmente reconocido/a de un niño. Una de las principales causas de la ausencia secundaria e involuntaria de hijos es la infertilidad.	Secondary involuntary childlessness
Ausencia voluntaria de hijos	Condición que describe a una persona que no desea o no ha deseado tener hijos y no tiene hijos biológicos, legales o reconocidos por la sociedad.	Voluntary childlessness
Azoospermia	La ausencia de espermatozoides en el eyaculado.	Azoospermia
Azoospermia no obstructiva	Ausencia de espermatozoides en el eyaculado debido a la falta de producción de espermatozoides maduros.	Non-obstructive azoospermia
Azoospermia obstructiva	Ausencia de espermatozoides en el eyaculado debido a la oclusión del sistema ductal.	Obstructive azoospermia
Baja respuesta a la estimulación ovárica (POR)	Condición en la que se desarrollan/obtienen menos de 4 folículos y/u ovocitos luego de la estimulación ovárica realizada para obtener más folículos y ovocitos.	Poor ovarian response (POR) to ovarian stimulation
Banco de embriones	Lugar donde se almacenan embriones criopreservados para su uso futuro.	Embryo bank
Banco de óvulos	Lugar donde se almacenan los óvulos criopreservados para su uso futuro.	Oocyte bank
Banco de semen	Lugar donde se almacenan espermatozoides criopreservados para su uso futuro.	Sperm bank
Binucleación	La presencia de dos núcleos en un blastómero (célula).	Binucleation
Blastocele	Región central del blastocisto llena de líquido.	Blastocoele
Blastocisto	Estado de desarrollo del embrión pre-implantacional que se produce en torno al día 5-6 tras la inseminación o la ICSI. El blastocisto contiene una cavidad central llena de líquido (blastocele), una capa externa de células (trofoectodermo) y un grupo interno de células (masa celular interna).	Blastocyst
Blastómero	Célula de un embrión en estado de clivaje.	Blastomere
Bloqueo hipofisario (*downregulation*)	Método farmacológico para impedir la liberación de gonadotrofinas (FSH, LH) de la hipófisis/glándula pituitaria.	Pituitary down- regulation
Calentamiento (células)	El proceso de elevar la temperatura de una célula/s vitrificada/s desde la temperatura de almacenamiento hasta la temperatura ambiente/fisiológica.	Warming (cells)
Células de la *corona* *radiata*	Las células más internas del *cumulus oophorus*.	Corona radiata cells
Célula de Leydig	Tipo de célula testicular situada en el espacio intersticial entre los túbulos seminíferos, que secreta testosterona.	Leydig cell
Célula de Sertoli	Tipo de célula no germinal del túbulo seminífero que media las acciones de la testosterona y de la hormona folículo estimulante (FSH) en el testículo, proporciona nutrientes y proteínas a las células espermatogénicas en desarrollo, crea la barrera hemato-testicular y secreta la hormona antimulleriana.	Sertoli cell
Ciclo de ART cancelado	Un ciclo de ART en el que se ha iniciado la estimulación o la monitorización ovárica con la intención de realizar un tratamiento, pero que no continuó con aspiración folicular o, en el caso de un embrión descongelado, no continuó con transferencia embrionaria.	Canceled ART cycle
Ciclo de ART con ovocitos congelados/descongelados	Procedimiento de ART en el que se realiza el seguimiento del ciclo con la intención de fecundar ovocitos descongelados y realizar una transferencia de embriones.	Frozen-thawed oocyte cycle
Ciclo de ART de receptoras	Ciclo de ART en el que una mujer recibe cigoto/s o embrión/embriones de donante/s o de su pareja.	Recipient ART cycle
Ciclo de congelación total	Ciclo de ART en el que, tras la aspiración de ovocitos, se criopreservan todos los ovocitos y/o embriones y no se transfieren ovocitos y/o embriones a una mujer en ese ciclo.	Freeze-all cycle
Ciclo de donación de ovocitos	Ciclo de ART en el que se recolectan ovocitos de una donante con fines reproductivos o de investigación.	Oocyte donation cycle
Ciclo de recepción de embriones	Ciclo de ART en el que el útero de una mujer se prepara para recibir uno o más embriones en estado de clivaje/blastocistos, resultantes de gametos que no proceden de ella ni de su pareja masculina, si está presente.	Embryo recipient cycle
Ciclo de recepción de espermatozoides	Ciclo de reproducción médicamente asistida (MAR) en el que una mujer recibe espermatozoides de una persona que no es su pareja sexual íntima. En el caso de un registro de ART, los datos de un ciclo de recepción de espermatozoides sólo quedarán registrados si se utilizan procedimientos de ART.	Sperm recipient cycle
Ciclo de recepción de ovocitos	Ciclo de ART en el que una mujer recibe ovocitos de una donante, o de su pareja en caso de mantener una relación homosexual, para utilizarlos con fines reproductivos.	Oocyte recipient cycle
Ciclo de transferencia de embriones	Ciclo de ART en el que uno o más embriones frescos o congelados/descongelados en estado de clivaje/blastocistos son transferidos al útero o a la trompa de Falopio.	Embryo transfer cycle
Ciclo de transferencia de embriones congelados/descongelados (FET)	Procedimiento de ART en el que se realiza un seguimiento del ciclo con la intención de transferir embrión/embriones congelado/s o vitrificado/s/descongelado/s a una mujer. Nota: Un ciclo de FET se inicia cuando se suministra la medicación específica o se inicia el monitoreo del ciclo en la receptora con la intención de transferir un embrión.	Frozen-thawed embryo transfer (FET) cycle
Ciclo iniciado de reproducción médicamente asistida (iMAR)	Un ciclo en el que la mujer recibe una medicación específica para la estimulación ovárica o en el que se lleva a cabo un monitoreo del ciclo con la intención de tratar, independientemente de que se realice o no la inseminación, de que se intente la aspiración folicular en un ciclo de estimulación ovárica o de que los óvulos o embriones se descongelen o se transfieran en un ciclo de transferencia de embriones congelados (FET).	Initiated medically assisted reproduction cycle (iMAR)
Ciclo natural de ART	Procedimiento de ART en el que se recolectan uno o más ovocitos de los ovarios durante un ciclo menstrual sin utilizar ningún compuesto farmacológico.	Natural cycle ART
Ciclo natural modificado	Procedimiento de ART en el que uno o más ovocitos se recolectan de los ovarios durante un ciclo menstrual natural. Los compuestos farmacológicos se administran con el único propósito de bloquear el aumento espontáneo de la hormona luteinizante (LH) y/o inducir la maduración final de los ovocitos.	Modified natural cycle
Cigoto	Célula única resultante de la fecundación de un óvulo por un espermatozoide y antes de que se complete la primera división mitótica.	Zygote
Cirugía reproductiva	Procedimientos quirúrgicos realizados para diagnosticar, conservar, corregir y/o mejorar la función reproductiva en hombres o mujeres. La cirugía con fines anticonceptivos, como la ligadura de trompas y la vasectomía, también se incluye dentro de este término.	Reproductive surgery
Compactación	Proceso durante el cual se forman uniones estrechas entre blastómeros yuxtapuestos que dan lugar a una masa sólida de células con membranas celulares indistinguibles.	Compaction
Concentración de espermatozoides	El número de espermatozoides en millones por ml de semen.	Sperm concentration
Concientización de la fertilidad	La comprensión de la reproducción, la fecundidad, la fecundabilidad y los factores de riesgo individuales relacionados (por ejemplo, la edad avanzada, los factores de salud sexual, como las infecciones de transmisión sexual, y los factores de estilo de vida, como el tabaquismo y la obesidad) y los factores de riesgo no individuales (por ejemplo, los factores ambientales y del lugar de trabajo); incluido el conocimiento de los factores sociales y culturales que afectan a las opciones para satisfacer la planificación reproductiva familiar, así como las necesidades de construcción familiar.	Fertility awareness
Conducto eyaculatorio	Tubo que pasa por la próstata, justo lateralmente al verumontanum, donde confluyen el conducto deferente y el conducto de la vesícula seminal.	Ejaculatory duct
Congelación lenta	Procedimiento de criopreservación en el que la temperatura de la/s célula/s se reduce de forma escalonada, normalmente a un ritmo controlado por computadora, desde la temperatura fisiológica (o ambiente) hasta la temperatura extremadamente baja.	Slow-freezing
Criopreservación	Proceso de congelación lenta o vitrificación para preservar el material biológico (por ejemplo, gametos, cigotos, embriones en estado de clivaje, blastocistos o tejido gonadal) a una temperatura extremadamente baja.	Cryopreservation
Criopreservación de óvulos	Congelación o vitrificación de óvulos para su uso futuro.	Oocyte cryopreservation
Criopreservación de tejido ovárico	Proceso de congelación lenta o vitrificación de tejido ovárico extirpado quirúrgicamente a fin de preservar la capacidad reproductiva.	Ovarian tissue cryopreservation
Criptorquidia	Testículo que no está en posición escrotal en el periodo neonatal y hasta, pero no limitado a, 1 año después del nacimiento. Si el testículo no ha descendido al escroto, esta afección puede causar una insuficiencia testicular primaria y un mayor riesgo de desarrollo de cáncer testicular.	Cryptorchidism
Cuerpos polares	Pequeños cuerpos celulares que contienen cromosomas segregados por el ovocito por división asimétrica durante la telofase. El primer cuerpo polar se extruye en la telofase I y normalmente contiene sólo cromosomas con cromátidas duplicadas (2c); el segundo cuerpo polar se extruye en respuesta a la fecundación o a la activación partenogenética y normalmente contiene cromosomas con cromátidas simples (1c).	Polar bodies
Cuidado de la fertilidad	Intervenciones que incluyen la concientización, el apoyo y el manejo de la fertilidad con la intención de ayudar a los individuos y a las parejas a cumplir sus deseos asociados con la reproducción y/o la formación de una familia.	Fertility care
*Cumulus oophorus*	La masa de varias capas de células de la granulosa que rodea al ovocito.	Cumulus oophorus
Defecto de la fase lútea	Anomalía poco definida del endometrio, presumiblemente debida a una secreción de progesterona anormalmente baja o a su acción sobre el endometrio.	Luteal phase defect
Densidad espermática	Medida de la relación masa/volumen (gravedad específica) de los espermatozoides.	Sperm density
Descongelación	Proceso que consiste en elevar la temperatura de la/s célula/s congelada/s lentamente desde la temperatura de almacenamiento hasta la temperatura ambiente/fisiológica.	Thawing
Desencadenamiento de la maduración ovocitaria	Intervención destinada a inducir a un ovocito *in vitro* o in vivo a reanudar la meiosis para alcanzar la madurez (es decir, para alcanzar la metafase II).	Oocyte maturation triggering
Detención de la espermatogénesis	Incapacidad de las células germinales para progresar a través de etapas específicas de la espermatogénesis al inicio o durante la meiosis.	Spermatogenic arrest
Diagnóstico y tamizaje (*screening*) genético pre-implantacional (PGD y PGS)	Estos términos han sido sustituidos por las pruebas genéticas pre-implantacionales (PGT) (véase el término PGT y sus definiciones).	Preimplantation genetic diagnosis (PGD) and screening (PGS)
Diploidía/euploidía	Condición en la que una célula tiene dos juegos de cromosomas haploides. Cada cromosoma de un juego está emparejado con su homólogo en el otro juego. Un embrión diploide tiene 22 pares de autosomas y dos cromosomas sexuales, la condición normal.	Diploidy/euploidy
Disfunción eréctil	Incapacidad de tener y/o mantener una erección suficiente para el coito.	Erectile dysfunction
Disminución de la espermatogénesis	Hallazgo histológico en el que la espermatogénesis está presente con pocas células en los túbulos seminíferos, lo que provoca una disminución o ausencia del número de espermatozoides en el eyaculado.	Decreased spermatogenesis
Disomía	Número normal de cromosomas caracterizado por 22 pares de cromosomas autosómicos y un par de cromosomas sexuales (XX o XY). El número de cromosomas en las células humanas es normalmente 46.	Disomy
Donación de embriones (para la reproducción)	Ciclo de ART, que consiste en la transferencia al útero o a las trompas de Falopio de una mujer receptora de un embrión resultante de gametos que no se originaron en la receptora femenina ni en su pareja masculina, si está presente.	Embryo donation (for reproduction)
Donación de ovocitos	El uso de ovocitos de una donante con fines reproductivos o de investigación.	Oocyte donation
Eclosión	Proceso por el que un embrión en el estado de blastocisto sale de la zona pelúcida y se separa de ella.	Hatching
Eclosión asistida	Procedimiento de ART en el que la zona pelúcida de un embrión se adelgaza o perfora mediante métodos químicos, mecánicos o láser.	Assisted hatching
Edad gestacional	Edad de un embrión o feto calculada mediante la mejor estimación obstétrica, determinada por evaluaciones que pueden incluir una ecografía temprana y la fecha de la última menstruación y/o detalles perinatales. En el caso de las gestaciones que resultan de ART, se calcula añadiendo 2 semanas (14 días) al número de semanas completas desde la fecundación. Nota: En el caso de los ciclos de transferencia de embriones congelados/descongelados (FET), la fecha estimada de fecundación se calcula restándole al día de la transferencia, el número combinado de días que un embrión estuvo en cultivo antes de la criopreservación y después de la descongelación.	Gestational age
Embarazo	Estado de reproducción que comienza con la implantación de un embrión en una mujer y termina con la expulsión y/o extracción completa de todos los productos de la implantación.	Pregnancy
Embarazo bioquímico	Embarazo diagnosticado únicamente por la detección de subunidad beta de gonadotrofina coriónica humana (hCG) en suero u orina.	Biochemical pregnancy
Embarazo clínico	Embarazo diagnosticado por la visualización ecográfica de uno o más sacos gestacionales o por signos clínicos definitivos de embarazo. Además del embarazo intrauterino, puede incluir un embarazo ectópico clínicamente documentado.	Clinical pregnancy
Embarazo clínico con actividad cardíaca fetal	Embarazo diagnosticado por ecografía o documentación clínica con al menos un feto con actividad cardíaca.	Clinical pregnancy with fetal heart beat
Embarazo de localización desconocida (PUL)	Un embarazo documentado por una prueba de hCG positiva sin visualización de embarazo por ecografía. Esta condición sólo existe cuando la concentración de hCG circulante es compatible con la visualización ecográfica de un saco gestacional.	Pregnancy of unknown location (PUL)
Embarazo ectópico	Embarazo fuera de la cavidad uterina, diagnosticado por ecografía, visualización quirúrgica o histopatología.	Ectopic pregnancy
Embarazo heterotópico	Gestación múltiple que incluye al menos un embrión implantado en la cavidad uterina y al menos un embrión implantado fuera de la cavidad uterina.	Heterotopic pregnancy
Embarazo intrauterino	Condición reproductiva en la que un embrión se ha implantado en el útero.	Intra-uterine pregnancy
Embrión	El organismo biológico resultante del desarrollo del cigoto, hasta 8 semanas completas después de la fecundación, lo que equivale a 10 semanas de edad gestacional.	Embryo
Embriones en estado de clivaje	Embriones a partir del estado de 2 células y hasta el estado de mórula, pero sin incluirla.	Cleavage stage embryos
Embrión postimplantacional	Embrión en un estado de desarrollo que va desde de la adherencia al endometrio hasta las 8 semanas completas después de la fecundación, lo que equivale a 10 semanas de edad gestacional.	Post-implantation embryo
Embrión pre-implantacional	Embrión en estado de desarrollo que comienza con la división del cigoto en 2 células y que termina justo antes de la implantación en el útero.	Pre-implantation embryo
Emisión (semen)	Contracciones coordinadas de los conductos deferentes, las vesículas seminales y los conductos eyaculadores que conducen a la deposición del semen en el meato uretral antes de la eyaculación.	Emission (semen)
Endometriosis	Enfermedad caracterizada por la presencia de epitelio y estroma similares al endometrio fuera del endometrio y el miometrio. La endometriosis intrapélvica puede localizarse superficialmente en el peritoneo (endometriosis peritoneal), puede extenderse 5 mm o más por debajo del peritoneo (endometriosis profunda) o puede presentarse como un quiste endometriósico ovárico (endometrioma).	Endometriosis
Epidídimo	Conducto enrollado en espiral que transporta los espermatozoides desde los testículos a través de los conductos eferentes hacia los conductos deferentes.	Epididymis
Espacio perivitelino	Espacio entre la membrana citoplasmática que encierra al ovocito y la capa más interna de la zona pelúcida (este espacio puede contener el primer y el segundo cuerpo polar y fragmentos extracelulares).	Perivitelline space
Espermatozoide	Célula reproductora masculina madura producida en los testículos que tiene la capacidad de fecundar a un óvulo. La cabeza transporta el material genético, la pieza media produce energía para el movimiento y la cola larga y delgada impulsa al espermatozoide.	Spermatozoon
Esterilidad	Estado permanente de infertilidad.	Sterility
Estimulación ovárica (OS)	Tratamiento farmacológico para inducir el desarrollo de los folículos ováricos. Puede ser utilizada con dos fines: 1) para el coito programado o la inseminación; 2) en las ART, para obtener múltiples ovocitos en la aspiración folicular.	Ovarian stimulation (OS)
Estimulación ovárica leve en la IVF	Protocolo en el que se estimulan los ovarios con gonadotrofinas, y/u otros compuestos farmacológicos, con la intención de obtener un número limitado de ovocitos después de la estimulación para la IVF.	Mild ovarian stimulation for IVF
Euploidía	Condición en la que una célula tiene cromosomas en un múltiplo exacto del número haploide; en el ser humano este múltiplo es normalmente dos. Así, un embrión normal que es euploide es también diploide.	Euploidy
Extracción de espermatozoides testiculares por microdisección (microTESE)	Procedimiento quirúrgico en el que se utiliza un microscopio operatorio para identificar los túbulos seminíferos que pueden contener espermatozoides los cuales se extraerán para IVF y/o ICSI.	Microdissection testicular sperm extraction (microTESE)
Eyaculación	Contracciones coordinadas del tracto genitourinario que conducen a la eyección de los espermatozoides y del fluido seminal.	Ejaculation
Eyaculación precoz	Condición en la que el semen se libera antes de lo deseado.	Premature ejaculation
Eyaculación retardada	Condición en que un hombre tarda un periodo prolongado de tiempo en alcanzar el orgasmo y la eyaculación.	Delayed ejaculation
Eyaculación *retardata*	Condición que resulta en la incapacidad de eyacular durante el coito vaginal.	Ejaculation retardata
Eyaculación retrógrada	Condición que provoca que el semen sea forzado a retroceder desde los conductos eyaculatorios hacia la vejiga durante la eyaculación.	Retrograde ejaculation
Fecundabilidad	La probabilidad de que un embarazo, durante un único ciclo menstrual en una mujer con una exposición adecuada al semen y sin anticoncepción, culmine en un nacido vivo. En estudios poblacionales, la fecundabilidad se mide frecuentemente como una probabilidad mensual.	Fecundability
Fecundidad	Clínicamente definida como la capacidad de tener un nacido vivo.	Fecundity
Fertilidad clínica	La capacidad de establecer un embarazo clínico.	Clinical fertility
Fertilidad	La capacidad de establecer un embarazo clínico.	Fertility
Fertilización (fecundación)	Secuencia de procesos biológicos que se inicia con la entrada de un espermatozoide al interior de un ovocito maduro, seguida de la formación de los pronúcleos.	Fertilization
Fertilización *in vitro* (IVF)	Secuencia de procedimientos que implica la fecundación extracorpórea de gametos. Incluye la inseminación *in vitro* convencional y la ICSI.	*In vitro* fertilization (IVF)
Feto	Estado de desarrollo de un organismo desde completadas las 8 semanas a partir de la fecundación (equivalentes a 10 semanas de edad gestacional) hasta el final del embarazo.	Fetus
Fragmentación del embrión	Proceso durante el cual uno o varios blastómeros liberan vesículas de membrana que contienen citoplasma y, en ocasiones, cromosomas enteros o cromatina.	Embryo fragmentation
Gestación múltiple	Un embarazo con más de un embrión o feto.	Multiple gestation
Gestación múltiple de alto orden	Un embarazo con tres o más embriones o fetos.	High-order multiple gestation
Globozoospermia	Describe espermatozoides que poseen un acrosoma reducido o ausente.	Globozoospermia
Haploidía	Condición en la que una célula tiene un set de cada uno de los 23 cromosomas. Los gametos humanos maduros son haploides, con sólo 23 cromosomas cada uno.	Haploidy
Hemorragia después de la aspiración de ovocitos	Hemorragia significativa, interna o externa, después de la recuperación de ovocitos por aspiración, que requiere hospitalización para transfusión de sangre, intervención quirúrgica, observación clínica u otro procedimiento médico.	Bleeding after oocyte aspiration
Hidrosalpinx	Trompa de Falopio ocluida distalmente, dilatada y con líquido en su interior.	Hydrosalpinx
Hiperespermia	Volumen elevado del eyaculado, por encima del límite superior de referencia. Al informar los resultados, deben especificarse los criterios de referencia.	Hyperspermia
Hipoespermatogénesis	Descripción histopatológica de una producción reducida de espermatozoides en los testículos.	Hypospermatogenesis
Hipogonadismo hipergonadotrópico	Falla gonadal asociada a una reducción en la gametogénesis, una disminución de la producción de esteroides gonadales y una elevada producción de gonadotrofinas.	Hypergonadotropic hypogonadism
Hipogonadismo hipogonadotrópico	Falla gonadal asociada a una reducción en la gametogénesis y en la producción de esteroides gonadales debido a una reducción en la producción o acción de las gonadotrofinas.	Hypogonadotropic hypogonadism
Hipospermia	Volumen de eyaculado inferior al límite inferior de referencia. Al informar los resultados, deben especificarse los criterios de referencia.	Hypospermia
Imágenes *time-lapse*	Grabación de secuencias fotográficas de imágenes microscópicas obtenidas a intervalos regulares en el laboratorio de ART, referidas a gametos, cigotos, embriones en estado de clivaje o blastocistos.	Time-lapse imaging
Implantación	Adherencia y posterior penetración en el endometrio de un blastocisto sin zona pelúcida, pero cuando se trata de un embarazo ectópico, ésto ocurre en un tejido fuera de la cavidad uterina. Este proceso se inicia entre 5 y 7 días después de la fecundación del ovocito y suele dar lugar a la formación de un saco gestacional.	Implantation
Inducción de la ovulación (OI)	Tratamiento farmacológico en mujeres con anovulación u oligoovulación a fin de inducir ciclos ovulatorios normales.	Ovulation induction (OI)
Infertilidad	Enfermedad caracterizada por la imposibilidad de establecer un embarazo clínico después de 12 meses de relaciones sexuales regulares y sin protección, o debido a un impedimento en la capacidad de reproducirse de una persona, ya sea como individuo o con su pareja. Las intervenciones en materia de fertilidad pueden iniciarse antes de un año en función de los antecedentes médicos, sexuales y reproductivos, la edad, los hallazgos físicos y las pruebas de diagnóstico. La infertilidad es una enfermedad que genera una discapacidad debida a un impedimento funcional.	Infertility
Infertilidad femenina	Infertilidad causada principalmente por factores femeninos que abarcan: alteraciones ovulatorias; reserva ovárica disminuida; anomalías anatómicas, endocrinas, genéticas, funcionales o inmunológicas del sistema reproductor; enfermedades crónicas; y condiciones sexuales incompatibles con el coito.	Female infertility
Infertilidad femenina primaria	Una mujer a la que nunca se le ha diagnosticado un embarazo clínico y que cumple con los criterios de ser clasificada como infértil.	Primary female infertility
Infertilidad femenina secundaria	Una mujer que no puede establecer un embarazo clínico pero que ha sido diagnosticada previamente con un embarazo clínico.	Secondary female infertility
Infertilidad inexplicable	Infertilidad en parejas con función ovárica, trompas de Falopio, útero, cérvix y pelvis aparentemente normales y con una frecuencia coital adecuada; y función testicular, anatomía genito-urinaria y eyaculación normales. La probabilidad de llegar a este diagnóstico depende de las metodologías utilizadas y/o disponibles.	Unexplained infertility
Infertilidad masculina	Infertilidad causada principalmente por factores masculinos que abarcan: parámetros o funciones anormales del semen; anomalías anatómicas, endocrinas, genéticas, funcionales o inmunológicas del sistema reproductor; enfermedades crónicas; y condiciones sexuales incompatibles con la capacidad de depositar el semen en la vagina.	Male infertility
Infertilidad masculina primaria	Un hombre que nunca ha generado un embarazo clínico y cumple con los criterios para ser clasificado como infértil.	Primary male infertility
Infertilidad masculina secundaria	Un hombre que no puede generar un embarazo clínico, pero que previamente ha generado un embarazo clínico.	Secondary male infertility
Inseminación de donante	Proceso de colocar en el tracto reproductivo de una mujer, espermatozoides procesados en el laboratorio o semen, proveniente de un hombre que no es su pareja sexual íntima, con el fin de iniciar un embarazo.	Donor insemination
Inseminación intracervical	Procedimiento en el cual los espermatozoides procesados en el laboratorio son colocados en el cuello del útero para intentar un embarazo.	Intra-cervical insemination
Inseminación intrauterina	Procedimiento en el cual los espermatozoides procesados en el laboratorio son colocados en el útero para intentar un embarazo.	Intra-uterine insemination
Inseminación *in vitro* convencional	La co-incubación *in vitro* de ovocitos con espermatozoides con el objetivo de que se produzca una fecundación extracorpórea.	Conventional *in vitro* insemination
Inseminación vaginal	Procedimiento en el que el semen, recogido en un preservativo no lubricado o por un método similar, se deposita en la cavidad vaginal de una mujer. Esta intervención puede ser realizada por la misma mujer que intenta un embarazo.	Vaginal insemination
Insuficiencia ovárica prematura	Condición caracterizada por un hipogonadismo hipergonadotrópico en mujeres menores de 40 años (también conocida como falla ovárica prematura o primaria). Incluye a mujeres con menopausia prematura.	Premature ovarian insufficiency
Insuficiencia testicular iatrogénica	Daños en la función testicular tras la radioterapia, la quimioterapia o el tratamiento hormonal; o desvascularización como consecuencia de una operación de hernia.	Iatrogenic testicular failure
Inyección intracitoplasmática de espermatozoides (ICSI)	Procedimiento en el que se inyecta un solo espermatozoide en el citoplasma del ovocito.	Intracytoplasmic sperm injection (ICSI)
Leucocitospermia	Número elevado de glóbulos blancos en el semen por encima del límite superior de referencia. Al informar los resultados, deben especificarse los criterios de referencia.	Leukospermia
Licuefacción del semen	Proceso por el que las enzimas proteolíticas degradan las proteínas provocando la licuefacción del plasma seminal.	Semen liquefaction
Maduración citoplasmática	Proceso en el cual el ovocito adquiere la capacidad de sustentar la maduración nuclear, la fecundación, la formación de pronúcleos, la singamia y las posteriores divisiones tempranas hasta la activación del genoma embrionario.	Cytoplasmic maturation
Maduración *in vitro* (IVM)	Secuencia de procedimientos de laboratorio que permiten la maduración extracorpórea de ovocitos inmaduros, generando ovocitos totalmente maduros capaces de ser fecundados con potencial para convertirse en embriones.	*In vitro* maturation (IVM)
Maduración nuclear	Proceso durante el cual el ovocito reanuda la meiosis y progresa de la profase I a la metafase II.	Nuclear maturation
Manejo expectante de la fertilidad	Manejo de los problemas de fertilidad, incluida la infertilidad, sin una intervención clínica o terapéutica específica, aparte de la información y consejería destinada a mejorar la probabilidad de lograr una gestación espontánea.	Expectant fertility management
Masa celular interna	Grupo de células adheridas al trofoectodermo polar formado por células madres embrionarias, que tienen el potencial de transformarse en células y tejidos del cuerpo humano, excepto la placenta o las membranas amnióticas.	Inner cell mass
Microdeleciones del cromosoma Y	Segmentos faltantes del material genético en el cromosoma Y que se asocian con anomalías en la espermatogénesis.	Y-chromosome microdeletions
Micromanipulación en ART	Procedimiento de ART realizado sobre el espermatozoide, el óvulo o el embrión; los procedimientos de micromanipulación más comunes en ART son la ICSI, la eclosión asistida y la biopsia de gametos o embriones para PGT.	Micromanipulation in ART
Monosomía	La ausencia de uno de los dos cromosomas homólogos en los embriones. Las monosomías autosómicas en los embriones no son compatibles con la vida. Los embriones con monosomías de cromosomas sexuales rara vez son compatibles con la vida.	Monosomy
Mortinato	La muerte de un feto antes de la expulsión o extracción completa después de finalizadas las 28 semanas de edad gestacional. La muerte se determina por el hecho de que, tras dicha separación, el feto no respira ni muestra ninguna otra evidencia de vida, como latidos cardíacos, pulsaciones del cordón umbilical o movimiento definido de los músculos voluntarios. Nota: incluye las muertes ocurridas durante el parto.	Stillbirth
Mórula	Embrión formado después de completar la compactación, normalmente 4 días después de la inseminación o la ICSI.	Morula
Mosaicismo	Trastorno en el que hay más de una población celular con distintos cariotipos que surgen de un solo embrión.	Mosaicism
Motilidad espermática	Porcentaje de espermatozoides mótiles en relación con el número total de espermatozoides.	Sperm motility
Muerte/mortalidad neonatal	Muerte de un bebé nacido vivo en los 28 días siguientes al nacimiento. Puede subdividirse en a) temprana, si la muerte se produce en los primeros 7 días después del nacimiento; y b) tardía, si la muerte se produce entre los 8 y los 28 días después del nacimiento.	Neonatal death/mortality
Muerte/mortalidad neonatal temprana	Muerte de un recién nacido en los 7 días siguientes al nacimiento.	Early neonatal death/mortality
Muerte/mortalidad perinatal	Muerte fetal o neonatal ocurrida al final del embarazo (a partir de 22 semanas completas de edad gestacional), durante el parto, o hasta 7 días completos después del nacimiento.	Perinatal death/mortality
Multinucleación	La presencia de más de un núcleo en una célula.	Multinucleation
Nacido (único)	La expulsión o extracción completa de un feto humano después de 22 semanas completas de edad gestacional, independientemente de que sea un nacimiento de un niño vivo o muerto, o si se desconoce la edad gestacional, un peso al nacer superior a 500 gramos. Un nacido único se refiere a un recién nacido individual; y un parto con múltiples nacidos, como un parto gemelar, se registraría como dos nacidos.	Birth (single)
Nacido a término	Nacimiento que tiene lugar entre las 37 y las 42 semanas completas de edad gestacional.	Full-term birth
Nacido extremadamente prematuro	Nacimiento que tiene lugar después de las 22 semanas, pero antes de las 28 semanas completas de edad gestacional.	Extremely preterm birth
Nacido postérmino	Nacimiento de un niño vivo o muerto que tiene lugar después de 42 semanas completas de edad gestacional.	Post-term birth
Nacido prematuro	Nacimiento que tiene lugar después de las 22 semanas y antes de las 37 semanas completas de edad gestacional.	Preterm birth
Nacido vivo	La expulsión o extracción completa de un producto de la fecundación, después de 22 semanas completas de edad gestacional; que después de dicha separación, respira o muestra cualquier otra evidencia de vida, como latidos cardíacos, pulsaciones del cordón umbilical o movimiento definido de los músculos voluntarios, independientemente de que el cordón umbilical haya sido cortado o de que la placenta se mantenga adherida. Si se desconoce la edad gestacional, puede utilizarse un peso al nacer igual o superior a 500 gramos. Los nacidos vivos se refieren al recién nacido individual; por ejemplo, un parto gemelar representa dos nacidos vivos.	Live birth
Necrozoospermia	Descripción de un eyaculado en el que no se encuentran espermatozoides vivos.	Necrozoospermia
Oligospermia	Término para describir el bajo volumen de semen que ahora se reemplaza por el término hipospermia para evitar la confusión con el término oligozoospermia.	Oligospermia
Oligozoospermia	Baja concentración de espermatozoides en el eyaculado por debajo del límite inferior de referencia. Al informar los resultados, debe especificarse el criterio de referencia.	Oligozoospermia
Oolema	La membrana citoplasmática que rodea al ovocito.	Oolemma
Ooplasma	El citoplasma del ovocito.	Ooplasm
Ovario poliquístico (PCO)	Ovario con al menos 12 folículos de 2-9 mm de diámetro (criterios de Rotterdam). La PCO puede estar presente en mujeres con síndrome de ovario poliquístico (PCOS), pero también en mujeres con una función ovulatoria normal y una fertilidad normal.	Polycystic ovary (PCO)
Ovocito	El gameto femenino.	Oocyte
Ovocito diándrico	Ovocito con un set extra de cromosomas haploides de origen paterno.	Diandric oocytes
Ovocito digénico	Ovocito con un set extra de cromosomas haploides de origen materno.	Digynic oocytes
Ovocito en maduración	Ovocito que ha pasado de la profase I pero no ha completado la telofase I, por lo que no presenta el primer cuerpo polar.	Maturing oocyte
Ovocito inmaduro	Ovocito en la profase de la meiosis I (por ejemplo, un ovocito en el estado de vesícula germinal (GV)).	Immature oocyte
Ovocito maduro (óvulo)	Ovocito en metafase de la meiosis II, que presenta el primer cuerpo polar y posee la capacidad de ser fecundado.	Mature oocyte
Ovulación	Proceso natural de expulsión de un óvulo de su folículo ovárico.	Ovulation
Padre(s) intencional(es)	Una pareja o persona que busca reproducirse con la ayuda de una portadora gestacional o una portadora gestacional tradicional.	Intended parent(s)
Partenote	El producto de un ovocito que se ha activado en ausencia del genoma paterno, de manera espontánea o con una intervención intencionada (activación inducida).	Parthenote
Parto	La expulsión o extracción completa de uno o más fetos de una mujer, después de al menos 22 semanas completas de edad gestacional, independientemente de que sean nacidos vivos o muertos. El parto de un solo recién nacido o de varios se considera un solo parto. Si se da a luz a más de un recién nacido, suele reconocerse como un parto con nacidos múltiples.	Delivery
Parto con nacidos múltiples luego de tratamientos de fertilidad	Un parto con más de un recién nacido, luego de cualquier tratamiento de fertilidad.	Delivery with multiple births after fertility treatments
Parto múltiple	La expulsión o extracción completa de más de un feto, después de 22 semanas completas de edad gestacional, independientemente de que sea un nacido vivo o muerto. Un parto de gemelos representa dos nacidos.	Multiple birth
Partos múltiples de alto orden	La expulsión o extracción completa de tres o más fetos, después de completadas las 22 semanas de edad gestacional, independientemente de que sean nacidos vivos o muertos.	High-order multiple births
Patología tubárica	Anormalidad tubárica que da lugar a una disfunción de la trompa de Falopio, incluida la obstrucción parcial o total de una o ambas trompas (proximal, distal o combinada), hidrosalpinx y/o adherencias peritubáricas y/o periováricas que afectan la función normal de captación ovocitaria. Suele ocurrir después de una enfermedad inflamatoria pélvica o de una cirugía pélvica.	Tubal pathology
Pequeño para la edad gestacional	Peso al nacer inferior al percentil 10% para la edad gestacional. Al comunicar los resultados, deben especificarse los criterios de referencia. Si se desconoce la edad gestacional, debe registrarse el peso al nacer.	Small for gestational age
Pérdida de embarazo	El resultado de cualquier embarazo que no resulta en al menos un nacido vivo. Al notificar la pérdida del embarazo, debe registrarse la edad gestacional estimada al final del mismo.	Pregnancy loss
Pérdida fetal	Muerte de un feto. Se denomina pérdida fetal temprana cuando la muerte tiene lugar entre las 10 y las 22 semanas de edad gestacional; pérdida fetal tardía, cuando la muerte tiene lugar entre las 22 y las 28 semanas de edad gestacional; y mortinato cuando la muerte tiene lugar después de las 28 semanas de edad gestacional.	Fetal loss
Perforación ovárica laparoscópica (drilling)	Un método quirúrgico que utiliza láser o electrocirugía para inducir la ovulación en mujeres con síndrome de ovario poliquístico anovulatorio u oligo-ovulatorio.	Laparoscopic ovarian drilling
Período neonatal	El período que comienza con el nacimiento y termina a los 28 días completos después del nacimiento.	Neonatal period
Peso bajo al nacer	Peso al nacer inferior a 2500 g.	Low birth weight
Peso extremadamente bajo al nacer	Peso al nacer inferior a 1000 g.	Extremely low birth weight
Peso muy bajo al nacer	Peso al nacer inferior a 1500 g.	Very low birth weight
Plasma seminal	Los fluidos del eyaculado.	Seminal plasma
Pobre respondedora en ART (POR)	Una mujer tratada con estimulación ovárica para ART, que presenta al menos 2 de las siguientes características: (1) edad materna avanzada (≥40 años); (2) una respuesta ovárica previa pobre (≤3 ovocitos con un protocolo de estimulación convencional destinado a obtener más de 3 ovocitos); y (3) una prueba de reserva ovárica anormal (por ejemplo, recuento de folículos antrales de 5-7 folículos u hormona antimulleriana de 0,5-1,1 ng/ml (criterios de Bolonia); u otros valores de referencia obtenidos de una población de referencia estandarizada).	Poor ovarian responder (POR) in assisted reproductive technology
Poliploidía	Condición en la que una célula tiene más de dos sets de cromosomas haploides: por ejemplo, un embrión triploide tiene tres sets de cromosomas y un embrión tetraploide tiene cuatro sets. La poliploidía en un embrión humano es incompatible con la vida.	Polyploidy
Polispermia	Proceso por el que un óvulo es penetrado por más de un espermatozoide.	Polyspermy
Portadora gestacional (gestante)	Mujer que lleva un embarazo con el acuerdo de que dará la descendencia a los padres intencionales. Los gametos pueden ser de los padres biológicos (intencionales) y/o de un/unos tercero/s. Éste reemplaza al término “sustituta gestacional”.	Gestational carrier
Portadora gestacional tradicional	Una mujer que dona sus ovocitos y es además la portadora gestacional de un embarazo resultante de fecundación de sus óvulos mediante un procedimiento de ART o una inseminación. Esto sustituye al término “sustituta gestacional tradicional”.	Traditional gestational carrier
Preservación de la fertilidad	Diversas intervenciones, procedimientos y tecnologías, incluida la criopreservación de gametos, embriones o tejidos ovárico y testicular para preservar la capacidad reproductiva.	Fertility preservation
Pronúcleo	Estructura redonda en el óvulo rodeada por una membrana que contiene cromatina. Normalmente, después de la fecundación se observan dos pronúcleos, cada uno de los cuales contiene un par de cromosomas haploides, uno procedente del óvulo y otro del espermatozoide, antes de la formación del cigoto.	Pronucleus
Prueba genética preimplantacional (PGT)	Prueba realizada para analizar el ADN de los óvulos (cuerpos polares) o de los embriones (estado de clivaje o blastocisto) para la determinación del tipo de antígenos leucocitarios humanos (HLA) o para determinar las anomalías genéticas. Éstas incluyen: PGT para aneuploidías (PGT-A); PGT para defectos monogénicos de un solo gen (PGT-M); y PGT para reordenamientos estructurales cromosómicos (PGT-SR).	Preimplantation genetic testing (PGT)
Quimerismo	Presencia de dos o más líneas celulares en un mismo individuo, cada una derivada de individuos diferentes.	Chimerism
Receptora (ART)	Persona o pareja que recibe óvulos, espermatozoides o embriones donados para iniciar un embarazo con la intención de convertirse en una madre y/o padre legalmente reconocido.	Recipient (ART)
Recuento total de espermatozoides	El número total de espermatozoides calculado en el eyaculado (volumen de semen multiplicado por la concentración de espermatozoides determinada a partir de una alícuota de semen).	Total sperm count
Reducción/desaparición espontánea de sacos gestacionales	La desaparición espontánea de uno o más sacos gestacionales con o sin embrión o feto en un embarazo múltiple documentado por ecografía.	Spontaneous reduction/vanishing sac(s)
Reducción embrionaria/fetal inducida	Intervención destinada a reducir el número de sacos gestacionales o de embriones/fetos en una gestación múltiple.	Induced embryo/fetal reduction
Reproducción médicamente asistida (MAR)	Reproducción llevada a cabo mediante diversas intervenciones, procedimientos, cirugías y tecnologías para tratar diferentes formas de trastornos de la fertilidad e infertilidad. Entre ellos se encuentran la inducción de la ovulación, la estimulación ovárica, el desencadenamiento de la ovulación, todos los procedimientos de ART, el trasplante uterino y la inseminación intrauterina, intracervical o intravaginal con semen del marido/pareja o de donante.	Medically assisted reproduction (MAR)
Reproducción póstuma	Proceso que utiliza gametos y/o embriones de una persona o personas fallecida/s con la intención de producir descendencia.	Posthumous reproduction
Reserva ovárica	Término generalmente utilizado para indicar el número y/o la calidad de los ovocitos, que es un reflejo de la capacidad reproductiva. La reserva ovárica puede evaluarse de varias maneras. Entre ellas se encuentran: la edad de la mujer; el número de folículos antrales en la ecografía; los niveles de la hormona antimulleriana; los niveles de la hormona folículo estimulante y del estradiol; la prueba de estímulo con citrato de clomifeno; la respuesta a la estimulación con gonadotrofinas, y la evaluación de ovocitos y/o embriones, basada en el número, la morfología o la evaluación genética de los mismos, durante un procedimiento de ART.	Ovarian reserve
Reserva ovárica disminuida	Término generalmente utilizado para indicar un número reducido y/o una calidad disminuida de ovocitos, de manera que la capacidad de reproducción se encuentra disminuida (ver reserva ovárica).	Diminished ovarian reserve
Respuesta ovárica excesiva	Respuesta exagerada a la estimulación ovárica caracterizada por la presencia de más folículos que los esperados. En general, se considera que más de 20 folículos > 12 mm de tamaño y/o más de 20 ovocitos recolectados tras la estimulación ovárica son excesivos, pero estos números son ajustables según diferentes variables, entre ellas étnicas.	Excessive ovarian response
Saco gestacional	Estructura llena de líquido asociada a un embarazo temprano, que puede estar situada dentro o, en el caso de un embarazo ectópico, fuera del útero.	Gestational sac
Salpingectomía	Extirpación quirúrgica de una trompa de Falopio completa.	Salpingectomy
Salpingitis ístmica nodosa (SIN)	Engrosamiento nodular de la trompa de Falopio proximal (donde las trompas se unen al útero), que puede deformar u ocluir las trompas y aumentar el riesgo de embarazo ectópico e infertilidad.	Salpingitis isthmica nodosa (SIN)
Salpingostomía	Procedimiento quirúrgico en el que se realiza una apertura en la trompa de Falopio para eliminar un embarazo ectópico o abrir una trompa obstruida (hidrosalpinx).	Salpingostomy
Semen/eyaculado	El fluido de la eyaculación, que contiene las células y secreciones procedentes de los testículos y de las glándulas sexuales accesorias.	Semen/Ejaculate
Simetría de blastómeros	El grado en que todos los blastómeros son uniformes en tamaño y forma.	Blastomere symmetry
Síndrome de células de Sertoli	Condición en la que sólo las células de Sertoli recubren los túbulos seminíferos y en la que suele haber una ausencia total de células germinales; también se denomina aplasia de células germinales. Excepcionalmente, puede observarse espermatogénesis en focos aislados.	Sertoli cell-only syndrome
Síndrome de hiperestimulación ovárica (OHSS)	Respuesta sistémica exagerada a la estimulación ovárica que se caracteriza por un amplio espectro de síntomas clínicos y manifestaciones de laboratorio. Puede clasificarse como leve, moderada o grave según el grado de distensión abdominal, el aumento de tamaño de los ovarios y las complicaciones respiratorias, hemodinámicas y metabólicas.	Ovarian hyperstimulation syndrome (OHSS)
Síndrome de hiperestimulación ovárica grave (OHSS)	Respuesta sistémica como resultado de la estimulación ovárica, que se caracteriza por molestias abdominales graves y/u otros síntomas de ascitis, hemoconcentración (Hct > 45) y/u otras anomalías bioquímicas graves que requieren hospitalización para observación y/o para intervención médica (paracentesis, otros).	Severe ovarian hyperstimulation syndrome (OHSS)
Síndrome de ovario poliquístico (PCOS)	Condición heterogénea, que requiere la presencia de dos de los siguientes tres criterios: (1) oligo-ovulación o anovulación; (2) hiperandrogenismo (evidencia clínica de hirsutismo, acné, alopecia y/o hiperandrogenemia bioquímica); (3) ovarios poliquísticos, evaluados mediante ecografía con más de 24 folículos antrales totales (de 2 a 9 mm de tamaño) en ambos ovarios.	Polycystic ovary syndrome (PCOS)
Singamia	Proceso durante el cual los pronúcleos femenino y masculino se fusionan.	Syngamy
Sobrepeso para la edad gestacional (macrosomía)	Peso al nacer superior al percentil 90% del peso al nacer, específico para el sexo y para una edad gestacional determinada de referencia. Para comunicar los resultados, deben especificarse los criterios de referencia. Si se desconoce la edad gestacional, debe registrarse el peso al nacer.	Large for gestational age
Soporte de la fase lútea	Suplementación hormonal en la fase lútea, generalmente de progesterona.	Luteal phase support
Subfertilidad	Término que debe utilizarse indistintamente con el de infertilidad.	Subfertility
Tasa de embarazo clínico	El número de embarazos clínicos expresado por cada 100 ciclos iniciados, aspirados o de transferencia embrionaria. Cuando se registran las tasas de embarazo clínico, se debe especificar el denominador (ciclos iniciados, aspirados o de transferencia embrionaria).	Clinical pregnancy rate
Tasa de fecundidad específica por edad (ASFR)	El número de nacidos vivos por mujer en un grupo de edad concreto en un año calendario específico, expresado por 1000 mujeres en ese grupo de edad.	Age specific fertility rate (ASFR)
Tasa de implantación	El número de sacos gestacionales observados, dividido por el número de embriones transferidos (normalmente expresado en porcentaje, %).	Implantation rate
Tasa de mortalidad neonatal	Número de muertes neonatales (hasta 28 días) por cada 1000 nacidos vivos.	Neonatal mortality rate
Tasa de mortalidad perinatal	El número de muertes perinatales por cada 1000 nacimientos totales (mortinatos más nacidos vivos).	Perinatal mortality rate
Tasa de mortinatos	El número de mortinatos por cada 1000 nacimientos totales (mortinatos más nacidos vivos).	Stillbirth rate
Tasa de nacimientos con anomalías congénitas	El número de nacimientos que presentan signos de anomalías congénitas por cada 10.000 nacimientos. Debe informarse el momento de la identificación.	Congenital anomaly birth rate
Tasa de parto	El número de partos expresado por cada 100 ciclos iniciados, ciclos de aspiración o ciclos de transferencia de embriones. Cuando se registran las tasas de partos, debe especificarse el denominador (ciclos iniciados, aspirados o de transferencia de embriones). Incluye los partos que dieron lugar a uno o más nacidos vivos y/o mortinatos. El parto de un embarazo único, gemelar o múltiple se registra como un solo parto. Si se da a luz a más de un recién nacido, suele reconocerse como un parto con nacidos múltiples.	Delivery rate
Tasa de parto acumulada por ciclo de aspiración/ciclo iniciado con al menos un nacido vivo	El número de partos con al menos un nacido vivo resultante de un ciclo de ART iniciado o aspirado, incluidos todos los ciclos en los que se transfieren embriones frescos y/o congelados, hasta que se produzca un parto con un nacido vivo o hasta que se utilicen todos los embriones, lo que ocurra primero. El parto de un nacido único, de un gemelo o de otros embarazos múltiples se registra como un solo parto. A falta de datos completos, se suele estimar la tasa de partos acumulada.	Cumulative delivery rate per aspiration/initiated cycle with at least one live birth
Tasa de parto con nacidos vivos	El número de partos que dieron lugar al menos a un nacido vivo, expresado por cada 100 ciclos. En el caso de las intervenciones de ART/MAR, pueden ser por ciclos iniciados, inseminados, aspirados o de transferencia de embriones. Cuando se indican las tasas de parto, debe especificarse el denominador (ciclos iniciados, inseminados, aspirados o de transferencia de embriones).	Live birth delivery rate
Tasa de parto por paciente después de un tratamiento de fertilidad	El número de partos con al menos un nacido vivo o un mortinato, expresado por cada 100 pacientes, después de un tiempo especificado y después de todos los tratamientos.	Delivery rate after fertility treatment per patient
Tasa total de fecundidad (TFR)	El número medio de nacidos vivos por mujer. Puede determinarse retrospectivamente, como datos observados (tasa total de fecundidad de la cohorte, CTFR) o como un número medio estimado (tasa total de fecundidad periódica, PTFR).	Total fertility rate (TFR)
Tasa total de fecundidad de la cohorte (CTFR)	El número medio observado de hijos nacidos vivos por mujer aplicado a una cohorte de mujeres de determinada edad, a través del tiempo. Se obtiene a partir de los datos de las mujeres después de finalizar su edad reproductiva.	Cohort total fertility rate (CTFR)
Tasa total de fecundidad por periodo (PTFR)	El número medio estimado de hijos nacidos vivos por mujer que nacerían de una cohorte de mujeres a lo largo de sus años reproductivos, si las tasas de fecundidad por edad en un período determinado permanecieron constantes en relación a la tasa de fecundidad específica por edad.	Period total fertility rate (PTFR)
Tasa total de partos con al menos un nacido vivo	El número total de partos con al menos un nacido vivo resultante de un ciclo de ART: iniciado o aspirado, incluyendo todos los ciclos en los que se transfieren embriones frescos y/o congelados, y más de un parto de un ciclo iniciado o aspirado si se produce, hasta que se utilicen todos los embriones. Notas: el parto de un nacido vivo único, gemelar o múltiple se registra como un solo parto. A falta de datos completos, la tasa total de partos se suele estimar.	Total delivery rate with at least one live birth
Tecnología de reproducción asistida (ART)	Todas las intervenciones que incluyen la manipulación *in vitro* de ovocitos, espermatozoides o embriones humanos con el fin de la reproducción. Esto incluye, entre otros, la IVF y la transferencia de embriones (ET), la inyección intracitoplasmática de espermatozoides (ICSI), la biopsia embrionaria, el testeo genético pre-implantacional (PGT), la eclosión asistida, la transferencia intratubárica de gametos (GIFT), la transferencia intratubárica de cigotos, la criopreservación de gametos y embriones, la donación de semen, ovocitos y embriones, y los ciclos de portadora gestacional. Por lo tanto, los registros de ART no incluyen la inseminación asistida con semen de la pareja de la mujer o de un donante de semen (Véase el término más amplio, reproducción médicamente asistida, MAR).	Assisted reproductive technology (ART)
Teratozoospermia	Porcentaje reducido de espermatozoides morfológicamente normales en el eyaculado por debajo de los límites inferiores de referencia. Al informarse los resultados, deben especificarse los criterios de referencia.	Teratozoospermia
Tiempo hasta el embarazo (TTP)	El tiempo que se tarda en lograr un embarazo, medido en meses o en número de ciclos menstruales.	Time to pregnancy (TTP)
Torsión ovárica	Rotación parcial o completa del pedículo vascular ovárico que provoca la obstrucción del flujo sanguíneo ovárico, pudiendo provocar la necrosis del tejido ovárico.	Ovarian torsion
Transferencia citoplasmática	Procedimiento que puede realizarse en diferentes etapas del desarrollo de un ovocito para añadir o sustituir diversas cantidades de citoplasma de un óvulo de donante.	Cytoplasmic transfer
Transferencia de pronúcleos	Transferencia de los pronúcleos del cigoto de una paciente a un cigoto donado que ha sido enucleado.	Pronuclei transfer
Transferencia del huso materno	Transferencia del huso materno (incluidos los cromosomas maternos) de un ovocito de la paciente a un ovocito donado en el que se ha eliminado el huso materno con cromosomas.	Maternal spindle transfer
Transferencia de un solo embrión (SET)	La transferencia de un embrión en un procedimiento de ART. Se considera electiva (eSET) cuando se dispone de más de un embrión de calidad suficiente para la transferencia.	Single embryo transfer (SET)
Transferencia electiva de embriones	La transferencia de uno o más embriones, seleccionados de una cohorte mayor de embriones disponibles.	Elective embryo transfer
Transferencia electiva de un solo embrión (eSET)	Transferencia de un embrión seleccionado de una cohorte mayor de embriones disponibles.	Elective single embryo transfer (eSET)
Transferencia embrionaria (ET)	Procedimiento por el cual se coloca en el útero un embrión en cualquier estado embrionario desde el día 1 al 7 después de la IVF o ICSI. Los embriones del día 1 al día 3 también pueden transferirse a la trompa de Falopio.	Embryo transfer (ET)
Transferencia embrionaria diferida	Procedimiento en el que la transferencia de embriones no se realiza dentro del plazo del ciclo de aspiración de ovocitos, pero en un momento posterior.	Delayed embryo transfer
Transferencia embrionaria doble (DET)	Transferencia de dos embriones en un procedimiento de ART. Puede ser electiva (eDET) cuando se dispone de más de dos embriones de calidad suficiente para la transferencia.	Double embryo transfer (DET)
Transferencia tubaria de cigotos (ZIFT)	Procedimiento de ART en el que se transfieren uno o más cigotos a la trompa de Falopio.	Zygote intrafallopian transfer (ZIFT)
Transferencia tubaria de gametos (GIFT)	Un procedimiento de ART en el que se transfieren ambos gametos (ovocitos y espermatozoides) a la/s trompa/s de Falopio.	Gamete intrafallopian transfer (GIFT)
Trisomía	Número anormal de copias cromosómicas en una célula que se caracteriza por la presencia de 3 cromosomas homólogos, en lugar de los 2 normales. La mayoría de las trisomías en embriones humanos son incompatibles con la vida.	Trisomy
Trofectodermo	Células que forman la capa exterior de un blastocisto y que tienen el potencial de convertirse en la placenta y en las membranas amnióticas.	Trophectoderm
Unisomía	Condición en una célula resultante de la pérdida de un solo cromosoma, originando una sola copia de ese cromosoma particular, en lugar de los 2 normales. La mayoría de las unisomías en embriones humanos son incompatibles con la vida.	Unisomy
Varicocele	Agrandamiento venoso en el plexo pampiniforme testicular.	Varicocele
Varicocelectomía	Procedimiento para ocluir o extirpar parte de la vena espermática interna en situaciones en las que se ha expandido en un varicocele.	Varicocelectomy
Vasectomía	Procedimiento para ocluir los conductos deferentes. Suele realizarse de forma bilateral para asegurar la esterilización.	Vasectomy
Vesícula germinal (GV)	El núcleo de un ovocito en la profase I.	Germinal vesicle (GV)
Viscosidad	La descripción de la fluidez relativa del semen.	Viscosity
Viscosidad del semen	La descripción de la fluidez relativa del plasma seminal.	Semen viscosity
Vitalidad de los espermatozoides	El porcentaje de espermatozoides vivos en relación con el número total de espermatozoides.	Sperm vitality
Vitrificación	Procedimiento de criopreservación ultrarrápido que evita la formación de hielo dentro de una célula cuya fase acuosa se convierte en un sólido similar al vidrio.	Vitrification
Volumen del semen	La cantidad de fluido en un eyaculado.	Semen volume
Zona pelúcida	Capa glicoproteica que rodea al ovocito.	Zona pellucida

We believe this Spanish version of the glossary will standardize and facilitate communication in the practice of ART not only in Spanish-speaking countries but also in countries where Spanish-speaking communities are visibly present. The Spanish translation of the glossary may be advantageous to Spanish-speaking minorities since it may attenuate language barriers, which are well recognized to have a negative impact on the quality of patients’ care (Al Shamsi *et al*., 2020). Furthermore, this translation will help patients and fertility specialists when confronted with the need of cross-border reproductive care (Nygren *et al*., 2010).
